# Double Two-State Opsin Model With Autonomous Parameter Inference

**DOI:** 10.3389/fncom.2021.688331

**Published:** 2021-06-16

**Authors:** Ruben Schoeters, Thomas Tarnaud, Luc Martens, Wout Joseph, Robrecht Raedt, Emmeric Tanghe

**Affiliations:** ^1^WAVES, Department of Information Technology (INTEC), Ghent University/IMEC, Ghent, Belgium; ^2^4BRAIN, Department of Neurology, Institute for Neuroscience, Ghent University, Ghent, Belgium

**Keywords:** computational optogenetics, computational efficiency, channelrhodopsin-H134R, MerMAID, model fitting

## Abstract

Optogenetics has a lot of potential to become an effective neuromodulative therapy for clinical applications. Selecting the correct opsin is crucial to have an optimal optogenetic tool. With computational modeling, the neuronal response to the current dynamics of an opsin can be extensively and systematically tested. Unlike electrical stimulation where the effect is directly defined by the applied field, the stimulation in optogenetics is indirect, depending on the selected opsin's non-linear kinetics. With the continuous expansion of opsin possibilities, computational studies are difficult due to the need for an accurate model of the selected opsin first. To this end, we propose a double two-state opsin model as alternative to the conventional three and four state Markov models used for opsin modeling. Furthermore, we provide a fitting procedure, which allows for autonomous model fitting starting from a vast parameter space. With this procedure, we successfully fitted two distinctive opsins (ChR2(H134R) and MerMAID). Both models are able to represent the experimental data with great accuracy and were obtained within an acceptable time frame. This is due to the absence of differential equations in the fitting procedure, with an enormous reduction in computational cost as result. The performance of the proposed model with a fit to ChR2(H134R) was tested, by comparing the neural response in a regular spiking neuron to the response obtained with the non-instantaneous, four state Markov model (4SB), derived by Williams et al. (2013). Finally, a computational speed gain was observed with the proposed model in a regular spiking and sparse Pyramidal-Interneuron-Network-Gamma (sPING) network simulation with respect to the 4SB-model, due to the former having two differential equations less. Consequently, the proposed model allows for computationally efficient optogenetic neurostimulation and with the proposed fitting procedure will be valuable for further research in the field of optogenetics.

## 1. Introduction

With optogenetics, neuronal firing can be controlled with light. This is achieved by genetically expressing opsins, light sensitive ion channels or pumps, in cells or cell subtypes. The merger of this genetic expression and optical stimulation results in superior spatiotemporal resolution with respect to the conventional neuromodulation techniques. Consequently, it is an ideal investigative tool for behavioral studies and a promising biomedical treatment for medical disorders such as epilepsy, Parkinson's disease and beyond the brain conditions (Aravanis et al., 2007; Abilez et al., 2011; Gerits and Vanduffel, 2013; Williams et al., 2013; Klapoetke et al., 2014; Carrette et al., 2015; Chen et al., 2015; Tønnesen and Kokaia, 2017).

The first light sensitive ion channels were discovered in the green alga *Chlamydomonas reinhardtii* by Nagel et al. (2002). Genetic engineering has led to a variety of opsins, such as red-shifted, step-function and ultrafast opsins, and mutants with altered ion selectivity (Gunaydin et al., 2010; Gerits and Vanduffel, 2013; Azimihashemi et al., 2014). An example of the latter is ChR2(H134R), which is addressed in this paper. Furthermore, other natural versions are continuously being discovered as well. An example are the MerMAIDs, which is a family of metagenomically discovered marine anion-conducting and intensely desensitizing channelrhodopsins (Oppermann et al., 2019).

**Figure 1:**
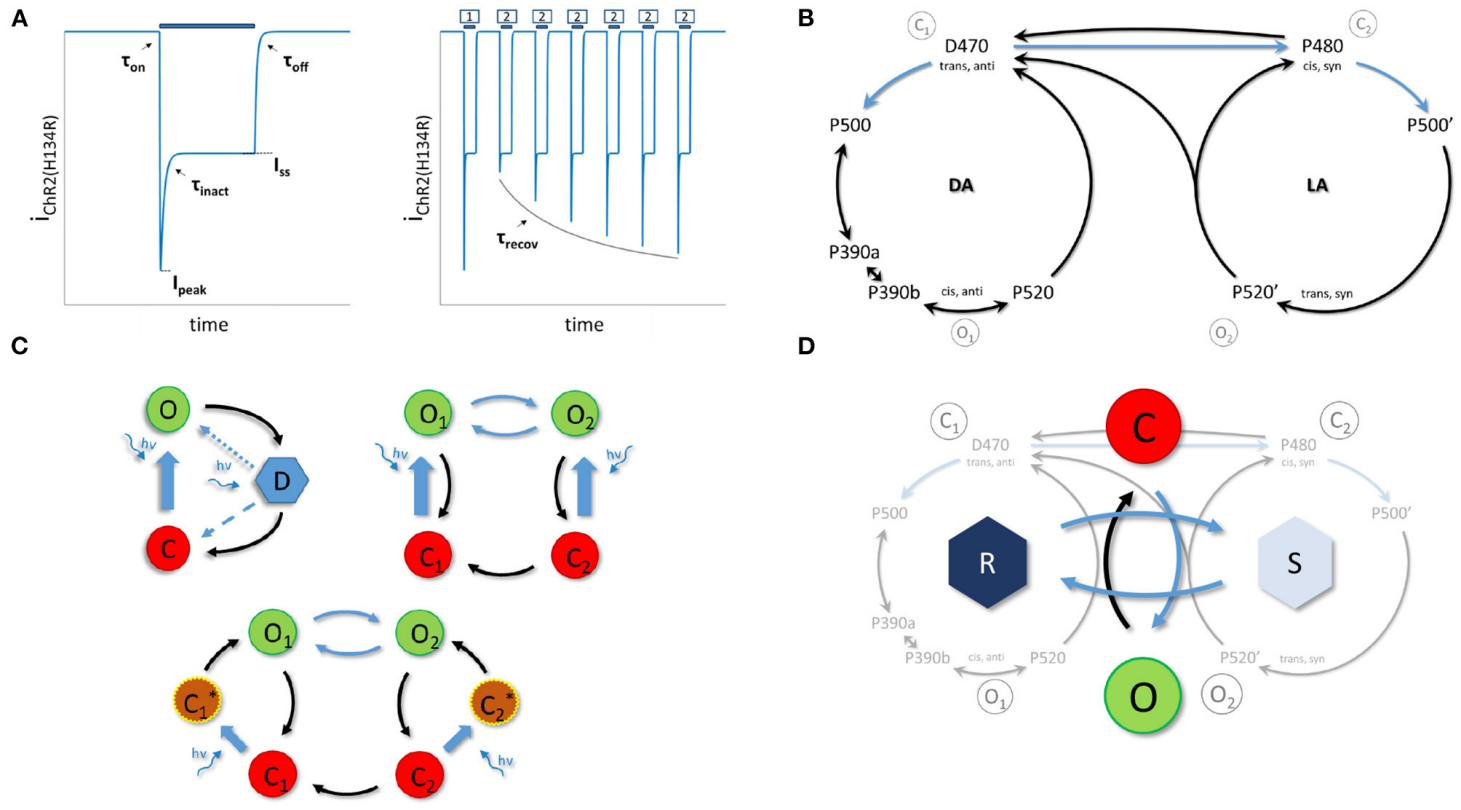
The Channelrhodopsin-2 photocurrent and photocycle. **(A)**, The photocurrent for a single light pulse on the left. Right, response to a S1-S2 pulse protocol with variable inter-pulse intervals. Light pulses are indicated with blue bars and target features with black arrows. **(B)**, The unified photocycle as proposed by Kuhne et al. (2019). **(C)**, Previously proposed models. (**C**, top left) a three state cycle model with second light dependent step (dotted or dashed step) (Ernst et al., 2008). (**C** top right) a four state branching model (Williams et al., 2013). (**C** bottom) a six state model with two extra activation intermediates (Grossman et al., 2013). **(D)**, The proposed double two-state opsin model (22OM) with separation of open-closing mechanism and conductance change due to dark-light adaptation. The latter is captured in the mathematical R and S model state pair. DA and LA indicate dark and light adapted molecule states, respectively. O means open, C is closed and D is desensitized. Blue arrows indicate light dependent rates.

In its initial dark-adapted (IDA) state and under voltage clamp conditions, ChR2's photocurrent exhibits a peak (I_*peak*_) and a steady-state current (I_*ss*_) (Bruun et al., 2015). The peak is reached within 1-2 ms and followed by fast decay onto a steady-state plateau. This is due to light adaptation (Figure 1A, left). Post-illumination, there is a bi-exponential decay back to baseline, rendering the channel in an apparent dark-adapted state (DA_*app*_). This is observed by applying a second stimulation after a short period of time (<10 s), which results in a reduced transient response with a maintained steady-state current (Figure 1A, right) (Nikolic et al., 2009; Bruun et al., 2015).

ChR2 comprises seven transmembrane helices. These are covalently bound with a retinal chromophore forming a protonated retinal Schiff base (RSBH^+^). In its IDA (D470), retinal is in an all-trans configuration (Bruun et al., 2015). Upon illumination, a 13 trans-cis isomerization is triggered that initiates a cascade of conformational changes with opening of the pore as result (P520). Before returning back to the dark adapted state, the channel converts to a non-conducting state P470. This happens on a millisecond timescale, while complete recovery takes seconds (Stehfest and Hegemann, 2010; Ritter et al., 2013; Volkov et al., 2017). There is strong evidence for a second photocycle, with similar intermediates (Stehfest and Hegemann, 2010; Schneider et al., 2013; Bruun et al., 2015; Deisseroth and Hegemann, 2017). However, the transition between the two photocycles is still under debate (Bruun et al., 2015; Deisseroth and Hegemann, 2017). Recently, Kuhne et al. (2019) proposed an unifying ChR2 photocycle model consisting of two parallel photocycles, with three reaction pathways (Figure 1B).

*In silico*, the photocurrent is currently modeled with either a three- or a four-state Markov model (Figure 1C). This is in accordance with the single and double photocycle hypothesis, respectively. The opening is reduced to a single state transition. This is because the D480 → P500 and P500 → P390 transitions occur on a much faster timescale. However, in order to represent fast closure, slow recovery and a steady-state current, a second photon absorption step is proposed for the three state model (Nagel et al., 2003; Stehfest and Hegemann, 2010). The photochemical transition either increases the recovery rate or acts as equilibrium modulator between the open and desensitized state. The six state model, as depicted in Figure 1C, bottom, is an extended version of the four state model. The additional two intermediates are to correctly account for the activation time after retinal isomerizations and to avoid explicit time dependent rates (Grossman et al., 2013). The four state model is in agreement with the second photocycle hypothesis with modeling of two open and closed states. The transition as depicted in Figure 1C, top right is according to the older transition hypothesis, not to the latest unifying photocycle model of Kuhne et al. (2019).

*In silico* studies, which allow for extensive and systematical investigation of the effects of the current kinetics, require an accurate model of the to be investigated opsin. To date, an accurate model consists of four differential equations (Williams et al., 2013). Using such a model therefore increases the computational burden enormously, especially in case of multi-compartment or network studies. Moreover, due to the expanding possibilities, selection of the correct opsin is crucial to have an optimal optogenetic tool. These four state Markov models are not easily fit as they require preliminary knowledge of the parameter space and its complex interactions. Furthermore, finding the optimal parameters is time-consuming as the set of differential equations has to be evaluated at each step in the selected optimization algorithm.

In this study we propose, for the first time (to the authors' knowledge), the use of a double two-state model structure for modeling of the opsin's photocurrents (Figure 1D). A fit is created of the ChR2(H134R) mutant and compared to the 4SB model of Williams et al. (2013). The performance of both models are tested in a regular spiking neuron (Pospischil et al., 2008). The difference in computation speed is assessed as well, this in the aforementioned regular spiking neuron for different stimulation patterns and in the sparse Pyramidal-Interneuron-Network-Gamma (sPING) network model (Börgers and Kopell, 2005; Sherfey et al., 2018) with increasing number of transfected neurons. Finally, the versatility of the proposed model is evaluated with a fit to a MerMAID opsin. The improvements of this work with respect to the current state of the art are:

- A double two-state opsin model structure resulting in a reduced complexity of fifty percent, which leads to an increase of computation speed valuable for optogenetic neurostimulation in conductance based models.
- A fitting procedure that allows for autonomous and accurate model fitting starting from a vast parameter space. Moreover, the fit time is reduced significantly by using an analytical solution to the set of differential equations (describing the double two-state model structure), possible under voltage clamp conditions and rectangular optical pulses.
- The proposed model structure can be used to accurately describe distinctive opsins.

However, the model does not include the non-instantaneous response of the retinal complex to light. Consequently, the response to short (< τ_on_) light pulses might be overestimated. Preliminary results of this work have been presented on the 28th Annual Computational Neuroscience Meeting (CNS*2019) (Schoeters et al., 2019).

## 2. Materials and Methods

In this study, a double two-state opsin model structure (22OM) was tested as alternative for opsin modeling. Below, we first describe the model in full and indicate the link between parameters and certain features. Next, the fitting procedure is elaborated. Finally, we describe the models and metrics used in the analysis of the model performance and computational speed.

### 2.1. The Model

The proposed model is based on the original voltage gated sodium model of Hodgkin and Huxley (Hodgkin and Huxley, 1952). It consists of two independent two-state pairs as depicted in Figure 1D. In contrast to the sodium model, where the second two-state pair represents the inactivation gate, it represents here the change in conductance due to dark-light adaptation.

After a long enough dark period, the molecules are assumed to be all in closed, dark adapted state. Upon stimulation, the channel opens with a transition C → O. On a slightly slower time scale the equilibrium between dark and light adapted molecules is reached. Light adapted molecules have a lower conductance than those that are in the dark adapted state. This change in conductance is captured by a transition R → S. The relationship between these mathematical model states and the physical dark and light adapted states of the opsin molecules is obtained via a linear transformation, i.e., *R* = (*g*_*ChR*2_·*DA* + *g*_*LA*_·*LA*)/*g*_*ChR*2_. Consequently, *R* (*S*) is one (zero) when fully dark adapted and *g*_LA_/*g*_ChR2_ (respectively, 1 − *g*_LA_/*g*_ChR2_) when fully light adapted, with *g*_LA_ the conductivity of a light adapted channel. *DA* and *LA* are the possibilities of the opsin molecules being in a dark or light adapted state, respectively. By using the R state in the model, *g*_LA_ does not need to be determined, therefore reducing the number of model parameters. The established equilibria of both state pairs depend on the level of optical excitation. After photostimulation, the channels close (O → C). Moreover, they all return to the dark adapted state after a long enough recovery period, which is on a much slower time scale than the other temporal kinetics. Because of this slower time scale, the transition S → R has to be light dependent as well. Otherwise the equilibrium would be completely on the side of S for every optical excitation level. The ChR2 photocurrent can thus be determined as follows:

(1) $$\begin{array}{l} i_{\text{ChR}2}=g_{\text{ChR}2}G\left(V\right)\;\left(O\cdot R\right)\;\left(V-E_{\text{ChR}2}\right) \end{array}$$

with

(2) $$\begin{array}{l} \frac{dO}{dt}=\frac{O_{\infty }\left(I,V\right)-O\left(t\right)}{\tau_{O}\left(I,V\right)} \end{array}$$

(3) $$\begin{array}{l} \frac{dR}{dt}=\frac{R_{\infty }\left(I,V\right)-R\left(t\right)}{\tau_{R}\left(I,V\right)} \end{array}$$

where *g*_ChR2_ is the maximal specific conductivity of the fully dark adapted channel, *G*(*V*) is a rectification function, *V* the membrane potential, *I* the light intensity, *E*_ChR2_ the equilibrium potential and *O* the fraction of molecules in the open state, with *O*_∞_ and τ_O_ its corresponding equilibrium and time constant. *R*_∞_ and τ_R_ are the respective equilibrium and time constants of the R state.

Under voltage clamp conditions and a rectangular optical pulse with constant light intensity, the photocurrent can be expressed in a closed form analytical expression:

(4) $$\begin{array}{l} i_{\text{ChR2}}=g_{\text{ChR2}}G(V)(O_{\text{ChR2}}^{\text{on}}(t)+O_{\text{ChR2}}^{\text{off}}(t))\cdot (R_{\text{ChR2}}^{\text{on}}(t)\\ \;+R_{\text{ChR2}}^{\text{off}}(t))(V-E_{\text{ChR2}}) \end{array}$$

with

(5) $$\begin{array}{l} O_{ChR2}^{\text{on}}\left(t\right)=\left[O_{\text{∞}}-\left(O_{\text{∞}}-O_{0}\right)\exp\left(-\frac{t-t_{\text{on}}}{\tau_{\text{O}}\left(I,V\right)}\right)\right]\\ \;\cdot \Theta \left(t-t_{\text{on}}\right)\Theta \left(t_{\text{off}}-t\right) \end{array}$$

(6) $$\begin{array}{l} O_{\text{ChR}2}^{\text{off}}\left(t\right)=O_{\text{ChR}2}^{\text{on}}\left(t_{\text{off}}\right)\exp\left(-\frac{t-t_{\text{off}}}{\tau_{\text{O}}\left(0,V\right)}\right)\Theta \left(t-t_{\text{off}}\right) \end{array}$$

(7) $$\begin{array}{l} R_{\text{ChR}2}^{\text{on}}\left(t\right)=\left[R_{\text{∞}}-\left(R_{\text{∞}}-R_{0}\right)\exp\left(-\frac{t-t_{\text{on}}}{\tau_{\text{R}}\left(I,V\right)}\right)\right]\\ \;\cdot \Theta \left(t-t_{\text{on}}\right)\Theta \left(t_{\text{off}}-t\right) \end{array}$$

(8) $$\begin{array}{l} R_{\text{ChR}2}^{\text{off}}\left(t\right)=\left[1-\left(1-R_{\text{ChR}2}^{on}\left(t_{\text{off}}\right)\right)\cdot \exp\left(-\frac{t-t_{\text{off}}}{\tau_{\text{R}}\left(0,V\right)}\right)\right]\\ \;\Theta \left(t-t_{\text{off}}\right) \end{array}$$

with Θ the Heaviside function, *O*_0_ and *R*_0_ the initial values of *O* and *R* at *t* = *t*_on_ (respectively, 0 and 1 when fully dark adapted) and, *t*_on_ and *t*_off_, respectively, the onset and offset of the optical pulse.

This is of particular use during the fitting procedure as the model is fit to experimental data, recorded under the same aforementioned conditions. Moreover, strong correlations between the model time constants and experimentally determined features (Figure 1A) are observed. These can be exploited to obtain a first approximation of the model's parameters (see section 2.2). When τ_O_ ≪ τ_R_, the transition rate time constant τ_O_ can be easily obtained from the activation (τ_on_) and deactivation (τ_off_) time constants. Under the same conditions, τ_R_ strongly correlates with the inactivation time constant (τ_inact_) when *I* ≠ 0. The recovery time constant needs to be scaled as shown in Equation 10 to get a good approximation of the dark-light adaptation time constant under dark (*I* = 0) conditions. This relationship is obtained by evaluating the recovery time definition with the given model equations, i.e., τ_recov_ = *t*_on,2_ − *t*_off,1_ → *I*_p,2_/*I*_p,1_ = 1 − exp(−1). Here, *t*_on,2_ is the onset time of the second pulse, *t*_off,1_ the offset of the first pulse, and *I*_p,2_ and *I*_p,1_ the current peak value of second and first pulse, respectively.

(9) $$\begin{array}{l} \tau_{\text{O}}\left(I,V\right)\approx \tau_{\text{on}},\tau_{\text{O}}\left(0,V\right)\approx \tau_{\text{off}}\text{ and }\tau_{\text{R}}\left(I,V\right)\approx \tau_{\text{inact}}\; \end{array}$$

(10) $$\begin{array}{l} \tau_{\text{R}}\left(0,V\right)\approx \tau_{\text{recov}}/\left(1-\ln\frac{1}{1-I_{\text{ratio}}}\right) \end{array}$$

Furthermore, following conditions need to be met for the relationship to hold true:

(11) $$\begin{array}{l} t_{\text{p},1}-t_{\text{on},1}\approx t_{\text{p},2}-t_{\text{on},2}\\ t_{\text{p},1}-t_{\text{on},1}>\tau_{\text{O}}\\ t_{\text{off},1}-t_{\text{on},1}>\tau_{\text{R}} \end{array}$$

The first, *t*_p,i_ − *t*_on,i_ is the time required to reach the peak value since onset of pulse i. This needs to be approximately the same in both first and second pulse, while these need to be significantly larger than the activation time constant. The last one requires that the steady-state value is reached at the end of the first pulse.

Unless specified, the time constants and time in this study are in seconds, the membrane potential in mV and the intensity in W/m^2^. The units of the conductance depend on the experimental data of each opsin, i.e., mS/cm^2^ and μS in case of the ChR2(H134R) and MerMAID fit, respectively.

### 2.2. The Fitting Procedure

Due to the dependency on both the potential and light intensity, more than twenty parameters need to be inferred. This vast parameter space impedes finding the optimal solution which is at a high computational cost. To alleviate this, the fitting procedure can be divided into four steps.

The first step is the extraction of the features, which is described by Williams et al. (2013). The peak current (*I*_peak_) is the maximal deflection from baseline. The steady-state current (*I*_ss_) is the plateau value. The current ratio (*I*_ratio_) is then *I*_ss_/*I*_peak_. The time constants are extracted using mono-exponential curve fits. To this end, a nonlinear least-squares curve fit is performed, with a trust-region-reflective algorithm. Furthermore, a multi-start algorithm with ten starting points was used to ensure finding of the global solution. The variable and function tolerance were set to 10^−12^. The recovery time constant, i.e., the time necessary between two pulses to have a second peak current which is 63% of the first peak (see definition in previous subsection), was determined from a set of two-pulse experiments.

Next, τ_O_ and τ_R_ are fit to the obtained target data. Both are fit to the corresponding time constants (see Equations 9, 10) using the aforementioned nonlinear least-squares method. Again, a multi-start algorithm is used but with 2000 starting points. For the intensity dependence, sigmoidal functions on the log-scale are used while for the voltage dependence a logistic regression was selected. The two dependencies are combined by either a multiplication or a reciprocal addition. The relationships and combination schemes are given by Equations 12–16, with p_i_, i = 1 → 6 indicating the unknown parameters of each relationship individually.

(12) $$\begin{array}{l} \tau_{\text{O}}\left(I\right)=\frac{p_{3}}{1+\exp\left(p_{1}/p_{2}\right)\cdot I^{1/p_{2}\cdot \ln\left(10\right)}} \end{array}$$

(13) $$\begin{array}{l} \tau_{\text{R}}\left(I\right)=p_{1}(1-\frac{p_{2}}{1+\exp\left(p_{3}/p_{4}\right)\cdot I^{-1/p_{4}\cdot \ln\left(10\right)}}\\ \;-\frac{\left(1-p_{2}\right)}{1+\exp\left(p_{5}/p_{6}\right)\cdot I^{-1/p_{6}\cdot \ln\left(10\right)}}) \end{array}$$

(14) $$\begin{array}{l} \tau_{\text{X}}\left(V\right)=\frac{p_{1}}{1+\exp\left(-\left(V-p_{2}\right)/p_{3}\right)} \end{array}$$

(15) $$\begin{array}{ll} \tau_{\text{X}}\left(I,V\right)=\; & \tau_{\text{X}}\left(I\right)\cdot \tau_{\text{X}}\left(V\right) \end{array}$$

or

(16) $$\begin{array}{l} {\left[{\left(\tau_{\text{X}}\left(I\right)\right)}^{-1}+{\left(\tau_{\text{X}}\left(V\right)\right)}^{-1}\right]}^{-1} \end{array}$$

(17) $$\begin{array}{l} O_{\text{∞}}\left(I\right)=\frac{1}{1+\exp\left(p_{1}/p_{2}\right)\cdot I^{-1/p_{2}\cdot \ln\left(10\right)}} \end{array}$$

(18) $$\begin{array}{l} R_{\text{∞}}\left(I\right)=1-\frac{p_{3}}{1+\exp\left(p_{1}/p_{2}\right)\cdot I^{-1/p_{2}\cdot \ln\left(10\right)}} \end{array}$$

(19) $$\begin{array}{l} G\left(V\right)=\frac{p_{1}\cdot \left(1-p_{2}\exp\left(-\left(V-E_{\text{ChR}2}\right)/p_{3}\right)\right)}{V-E_{\text{ChR}2}} \end{array}$$

In a third step, the parameters of the rectification function *G*(*V*) and the equilibrium constants *O*_∞_ and *R*_∞_ are fit. The used relationships are given in Equations 17–19, respectively. The potential dependence of *O*_∞_ and *R*_∞_ are omitted because this is mostly covered by the rectification function. The parameter values are determined by minimizing the cost function described below:

(20) $$\begin{array}{l} f_{\text{cost}}={\left(\frac{1}{N}\left[\underset{i=1\to N}{\sum }\Delta I_{\text{peak}}{\left(I_{i},V_{i}\right)}^{2}+\Delta I_{\text{ss}}{\left(I_{i},V_{i}\right)}^{2}+\Delta I_{\text{ratio}}{\left(I_{i},V_{i}\right)}^{2}\right]\right)}^{1/2}\\ \;\Delta I_{\text{x}}\left(I_{i},V_{i}\right)=w_{\text{x}}\left(y_{\text{x}}\left(I_{i},V_{i}\right)-t_{\text{x},{\text{I}}_{\text{i}},{\text{V}}_{\text{i}}}\right),\text{with }x=peak,ss,ratio \end{array}$$

Here, *y*_x_ and *t*_x_,_I_i_,V_i__ are, respectively the model output and target value at stimulation values (*I*,*V*), with $y_{\text{peak}}=\max_{\text{t}}\left(\left|i_{\text{ChR}2}^{\text{on}}\left(t,I,V\right)\right|\right)$, $y_{\text{ss}}=i_{\text{ChR}2}^{\text{on}}\left(t_{\text{off}}\right)$ and *y*_ratio_ = *y*_ss_/*y*_peak_. $i_{\text{ChR}2}^{\text{on}}\left(t,I,V\right)$ is the current during the photostimulation pulse (*t ϵ* [*t*_on_, *t*_off_]) for a certain intensity *I* and voltage *V*. The current is calculated by evaluating Equations 4–8 with the determined dependencies in the previous step. *N* is the total number of stimulation sets (*I*,*V*). The minimization of *f*_cost_ is performed with the MATLAB *fmincon*-function and multi-start algorithm with 3000 starting points to increase chance of finding the global optimum. The upper and lower boundaries as well as the initial conditions are summarized in Table 1. Extra nonlinear constraints are applied to assure that *O*_∞_ approaches one for high intensities (see sections 3.1, 3.4) and *G*(*V*) ≥ 0. A final constraint ensures a current decay back to baseline after the optical stimulation, i.e., *i*_on_(*t*_off_) > *i*_off_(*t*) or $O^{\text{on}}\left(t_{\text{off}}\right)\cdot R^{\text{on}}\left(t_{\text{off}}\right)>O^{\text{off}}\left(t\right)\cdot R^{\text{off}}\left(t\right)$, resulting in:

(21) $$\begin{array}{l} R_{\text{∞}}\left(I,V\right)>1-\frac{\tau_{\text{R}}\left(0,V\right)}{\tau_{\text{R}}\left(0,V\right)+\tau_{\text{O}}\left(0,V\right)} \end{array}$$

Finally, a global optimization is performed with the parameters of all rate functions included. First, a new parameter space is defined, which is 10% of the original parameter space but centered around the values obtained in previous steps and limited by the former. With the gathered dependencies, the ChR2 current is calculated according to Equation 4. All model features are now extracted in the same manner as performed on the experimental data. These are used to determine a cost function which is the weighted root mean square error Equation 20, with additional terms: $\Delta \tau_{\text{on}}{\left(I,V\right)}^{2}$, $\Delta \tau_{\text{off}}{\left(I,V\right)}^{2}$, $\Delta \tau_{\text{inact}}{\left(I,V\right)}^{2}$ and $\Delta \tau_{\text{rec}}{\left(I,V\right)}^{2}$. Subsequently, the problem is optimized with a bounded particle swarm optimization (Hassan et al., 2005; Helwig and Wanka, 2007; Chen, 2018), containing 1000 particles and with a time limit of 24 h. The same solver settings and constraints are imposed as described in previous steps. The single-pulse experiments are evaluated with a time step of 1.5·10^−4^s, while for the two-pulse experiments a step of 1 ms is used.

**Table 1:** Summary of all parameters.

	τ_O_(I)			τ_O_(V)			τ_R_(I)						τ_R_(V)		
	p_1_	p_2_	p_3_	p_1_	p_2_	p_3_	p_1_	p_2_	p_3_	p_4_	p_5_	p_6_	p_1_	p_2_	p_3_
LB	–10	0	0	0	–100	–1,000	0	0	–10	0	–10	0	0	–100	–1,000
UB	10	20	1	100	100	1,000	10	1	10	20	10	20	100	100	1,000
X0	1	1	0.5	1	-50	10	1	0.5	0	0.125	3	0.5	1	–50	10
RSRS-intm.	1.93	0.68	0.022	23.26	0.14	12.40	10	0.56	–1.59	0.88	1.96	0.11	100	–38.94	14.70
RSRS-final	1.81	1.17	0.021	23.14	–0.39	13.19	10	0.56	–1.58	0.87	1.96	0.11	99.74	–38.69	12.02
PP-intm.	1.99	0.67	0.034	0.64	–89.16	14.31	6.74	0.50	2.00	0.11	–1.3	0.88	1.50	–70.01	19.13
PP-final	1.93	0.88	0.030	0.63	-88.67	8.37	6.73	0.50	1.98	0.11	–1.28	0.88	1.66	–64.54	28.55
MM-intm.	1.70	1.49	0.035	0.29	48.56	738.24	0.17	0.0081	–2.80	14.69	1.05	0.42	24.77	80.92	164.75
MM-final	3.70	3.35	0.037	0.20	49.99	718.60	0.18	0.0082	–3.00	15.57	0.998	0.429	24.42	80.87	172.82
		O_∞_(I)			R_∞_(I)				G(V)				g_ChR2_	E_ChR2_	
		p_1_	p_2_		p_1_	p_2_	p_3_		p_1_	p_2_	p_3_				
LB		–10	0		–10	0	0|0.8		0|-	1.1|-	0|-		-|0	-|–100	
UB		10	20		10	20	1		100|-	100|-	500|-		-|100	-|100	
X0		1	1		1	1	0.1|0.9		1|-	10|-	50|-		-|30	-|0	
RSRS-intm.		3.45	0.71		2.03	0.13	0.71		9.91 (1)	1.24	46.17		1 (9.91)	0	
RSRS-final		3.38	0.62		1.96	0.12	0.77		10.77 (1)	1.25	44.52		1 (10.77)	0	
PP-intm.		3.45	0.71		1.99	0.15	0.73		8.93 (1)	1.27	42.37		1 (8.93)	0	
PP-final		3.44	0.68		2.25	0.065	0.75		9.10 (1)	1.27	41.47		1 (9.10)	0	
MM-intm		3.76	0.40		0.74	0.52	1.00		-	-	-		62.00	-3.64	
MM-final		3.67	0.39		0.40	0.54	0.9987		-	-	-		62.22	-3.62	

*The boundary conditions, lower bounds (LB) and upper bounds (UB), of first 2 steps of the fitting procedure and initial values (X0). The intermediate (-intm) and final (-final) model parameters of the selected mutant and model, with RSRS the H134R mutant with a double reciprocal addition combination of time constant dependencies (τ_X_(I) and τ_X_(V), Equation 16), PP the H134R mutant with a double product combination of time constants dependencies Equation 15 and the MerMAID fit (MM). Parameters that varied between ChR2(H134R) and MM fit are separated with vertical line. Between brackets is another parameter solution, resulting in the same model*.

### 2.3. Performance Tests

In this study, two opsin fits were performed. First, a fit is made to the data reported by Williams et al. (2013) of the ChR2(H134R) (Williams et al., 2013). The model accuracy is compared to the four state Markov model created by the same group. Four metrics are used to analyze the goodness-of-fit, i.e., Root mean square error (RMSE), Root mean square normalized error (RMSNE), Root mean square weighted error (RMSWE) and root mean square Z-score error (RMSZE):

(22) $$\begin{array}{l} RMSWE={\left(\frac{1}{N}\underset{i=1\to N}{\sum }w_{x}^{2}\cdot {\left[y_{x}\left(I_{i},V_{i}\right)-t_{x,I_{i},V_{i}}\right]}^{2}\right)}^{1/2} \end{array}$$

where *w*_x_ equals 1, 1/*t*_x_,_I_i_,V_i__, or 1/σ_x_,_I_i_,V_i__ in case of RMSE, RMSNE or RMSZE, respectively. *y*_x_(*I*_*i*_, *V*_*i*_), *t*_x_,_I_i_,V_i__ and σ_x_,_I_i_,V_i__ are the model output, target feature and standard deviation of target feature x under intensity *I* and voltage *V* of set i, and *w*_x_ are the weights used in *f*_cost_. The metrics are also determined in the overall, time constant features (τ_on_ + τ_off_ + τ_inact_ + τ_rec_) only and current features (*I*_p_ + *I*_ss_ + *I*_ratio_) only case. Here the squared errors of all features are summed first before taking the root and mean. The RMSWE is equivalent to the training error. However, it could not be used to compare the model fits as the used weights were not equal across fitting procedures (different weights were used in the 4SB fit, see Williams et al., 2013). Therefore, the other metrics were defined as well. Where the RMSE is biased by high values, the RMSNE is biased by values close to zero and RMSZSE which includes the uncertainty of the target features via σ_x_,_I_i_,V_i__ but could not be determined for the recovery time constant.

Both models are then implemented in a regular spiking neuron, described in Pospischil et al. (2008). The strength duration curves (SDC) are determined. When the irradiance is selected as strength for the SDC, a poor fit is obtained. This is due to the assumption of an RC equivalent circuit and a rectangular stimulation pulse in the Hill-Lapicque relationship Equation 23 (Noble and Stein, 1966; Williams and Entcheva, 2015). Therefore, the SDC fit is performed on the average inward stimulation current or temporal averaged current (*i*_ChR2,avg_, TAC), as described by Williams and Entcheva (2015).

(23) $$\begin{array}{l} i_{\text{ChR}2,\text{avg}}=\frac{I_{\text{ChR}2,\text{rheo}}}{\left(1-\exp\left(-\frac{PD}{\tau_{\text{ChR}2,\text{chron}}/\ln\left(2\right)}\right)\right)} \end{array}$$

(24) $$\begin{array}{l} i_{\text{ChR}2,\text{avg}}=\frac{1}{PD}\cdot \int_{0}^{T_{\text{end}}}i_{\text{ChR}2}\left(t\right)dt \end{array}$$

with PD the pulse duration and *T*_end_ one second after the end of the pulse. The relationship between the irradiance and *i*_ChR2,avg_ is obtained through a power series fit, which allows calculation of the irradiance rheobase (*I*_rheo_) and chronaxie (τ_chron_) as follows:

(25) $$\begin{array}{l} I_{\text{rheo}}=a\cdot I_{\text{ChR}2,\text{rheo}}^{b}+c \end{array}$$

(26) $$\begin{array}{l} \tau_{\text{chron}}=-\frac{\tau_{\text{ChR}2,\text{chron}}}{\ln\left(2\right)}\ln\left(1-\frac{I_{\text{ChR}2,\text{rheo}}}{{\left[\left(2I_{\text{rheo}}-c\right)/a\right]}^{1/b}}\right) \end{array}$$

where a, b and c are parameters obtained in an empirically power series fit of the irradiance curve vs. the inward stimulation current ($I=a\cdot {\left(i_{\text{ChR}2,\text{avg}}\right)}^{b}+c$) (Williams and Entcheva, 2015).

Moreover, the simulation speed is determined for different stimulation paradigms, i.e., simulation time (*T*_end_)/runtime in a regular spiking neuron (Pospischil et al., 2008). Therefore, we varied the pulse repetition frequency, stimulation time and duty cycle. The intensity was fixed for each model and set to a value that elicited a firing rate of 100 Hz in the regular spiking neuron in case of a two pulse stimulation of 2 s with duty cycle 0.5 and pulse repetition frequency of 1 Hz. The models were solved by the MATLAB Variable Step Variable Order solver (VSVO) ode113-solver (order 1-13, Adams-Bashort-Moulton predictor-corrector pairs) (Shampine and Reichelt, 1997), with a maximum time step of 100 μs and default tolerances, i.e., relative and absolute tolerance equal to 10^−3^ and 10^−6^, respectively.

Finally, computational gain with the proposed model compared to the 4 state Markov model was tested in a network model with an increasing number of transfected neurons. Therefore, we used the sparse Pyramidal-Interneuron-Network-Gamma (sPING) (Börgers and Kopell, 2005), which was implemented via the DynaSim toolbox (Sherfey et al., 2018). The ChR2(H134R) models were added to the pyramidal neurons. The number of inhibitory neurons was varied between 3 and 100 while the 4/1, pyramidal/interneuron-ratio was maintained. The network was fully connected and the GABAa and AMPA conductivities were scaled such that the total input per neuron stayed the same, i.e., $g_{\text{GABAa}}=2/\left(N_{\text{intern}}\right)\left[\text{mS}/\text{c}{\text{m}}^{2}\right]=g_{\text{AMPA}}$, with *N*_intern_ the number of interneurons in the sPING-network. In each case a single pulse stimulation of 300 ms was applied with a total simulation time of 500 ms. The irradiance was set such that the firing rates were equal for both ChR2 models. The study was performed with both a fixed step (10 μs) runge-kutta 4 solver and an ode15s-solver (stiff VSVO-solver, order 1-5, based on numerical differentiation formulas) (Shampine and Reichelt, 1997) with a maximum time step of 100 μs, and a relative and absolute tolerance of 10^−6^.

The results shown in this paper are computed with a 3.4 GHz clock rate, quad core system and 8 GB RAM.

### 2.4. Versatility

The versatility of the proposed model structure is shown with a fit to the MerMAID1 opsin (Oppermann et al., 2019). For more detail on the data set, we refer to the work of Oppermann et al. (2019). The same metrics as aforementioned are used to assess the fit accuracy.

## 3. Results

To test the feasibility of the proposed double two-state opsin model structure (22OM), it was fit to two data sets. First, we fitted the model to the data set of a ChR2(H134R) opsin reported by Williams et al. (2013), which was collected in a ChR2(H134R)-HEK293 stable cell line (Williams et al., 2013). By the same group already a four state Markov model was fit. This allowed us to analyze the performance of our model in detail. To this end, a comparison of the response to optical stimuli was made in a regular spiking neuron (Pospischil et al., 2008). Moreover, the computational speed was determined for different stimulation paradigms in the former neuron model as well as in the sPING (Börgers and Kopell, 2005) network model with increasing number of transfected neurons. Finally the versatility of the proposed modeling scheme was assessed with a fit to a MerMAID opsin which is an anion-conducting and intensely desensitizing channelrhodopsin.

### 3.1. The ChR2(H134R) Fit

A 22OM fit of the ChR2(H134R) opsin was obtained by applying the fitting procedure, described in the materials and methods section 2.2, to the experimental data. As Williams et al. (2013) already reported the target features, the first step could be omitted. The absence of differential equations in our fitting procedure allowed for multiple fits to be made, due to the significant reduction of the computational cost. Multiple weight sets, non-linear constraints and combinations of dependency addition of the time constants (product Equation 15 and reciprocal sum Equation 16) were tested. The parameters of the two best fits are shown in Table 1, where RSRS and PP is the fit with a double reciprocal sum and product combination, respectively. Both results were obtained with *w*_peak_ = 10, *w*_ss_ = 20, *w*_ratio_ = 50, *w*_on_ = 1000, *w*_inact_ = 1000, *w*_off_ = 1000, *w*_recov_ = 20, and a constraint where *O*_∞_(*I, V*) > 0.6 for *I* ≥ 5500 W/m^2^. The weights are chosen as such to level the differences between features to the same order of magnitude. As a result, all features have the same impact in the cost function with a slight preference for the current features. The time constant features are all expressed in seconds, while their values are in the order of milliseconds (except τ_recov_), explaining the high weight values. The extra constraint is justified as the current peak already starts to saturate for the highest intensity values, thus clamping the intensity dependence of the open steady-state value above the bending point in the logistics curve.

**Figure 2:**
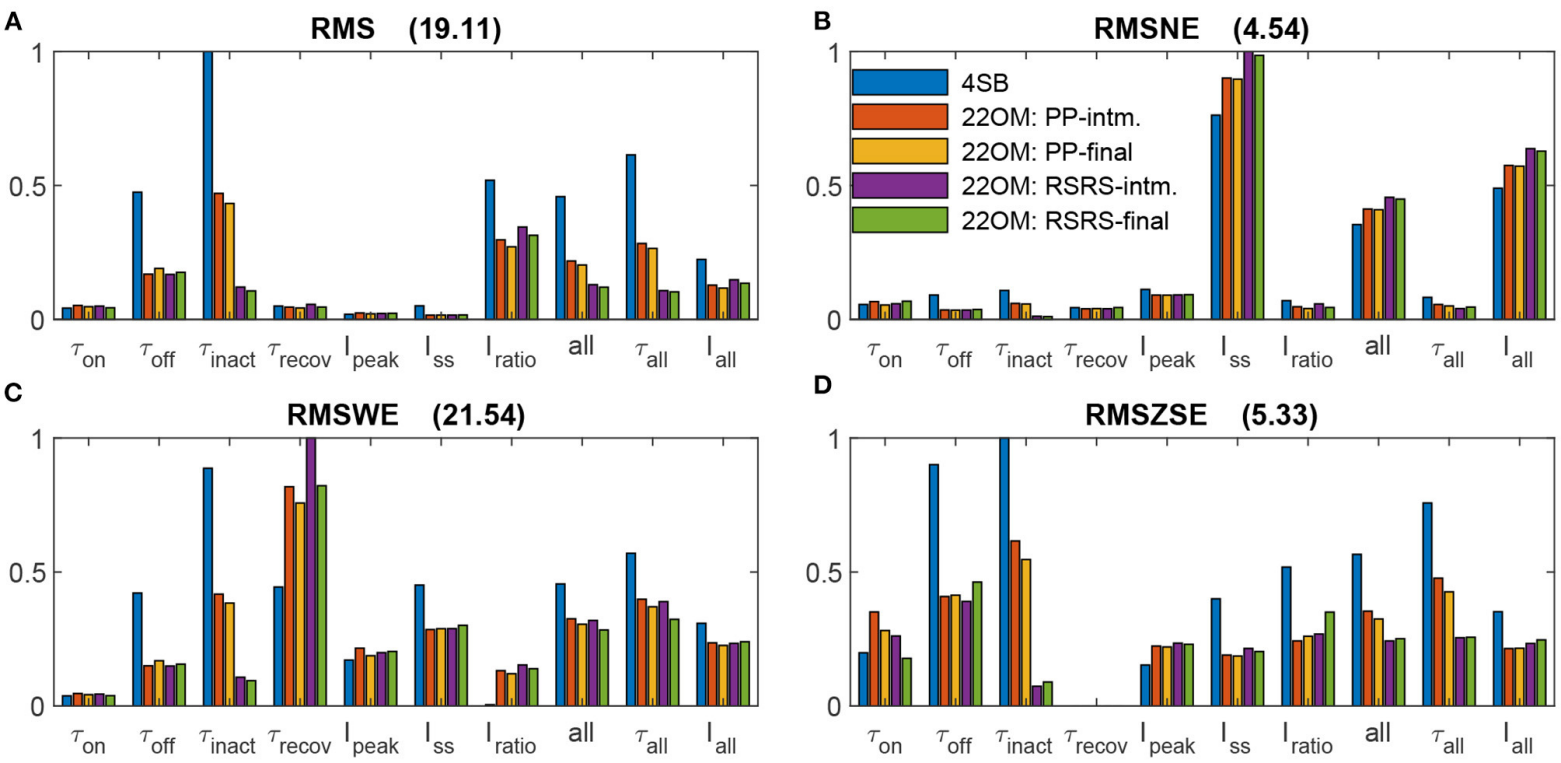
Normalized goodness-of-fit results of the model fits to the ChR2(H134R) data reported by Williams et al. (2013). Goodness-of-fit according to four metrics: Root-mean-square error **(A)**, Root-mean-square normalized error **(B)**, Root-mean-square weighted error **(C)** and Root-mean-square of z-score error **(D)**. The compared models are the 4SB model reported by Williams et al. (2013), the 22OM model with twice the product combination of time constants (intermediate fit 22OM: PP-intm and final fit 22OM: PP-final), and the 22OM model with twice the reciprocal addition combination of time constants (intermediate fit 22OM: RSRS-intm and final fit 22OM: RSRS-final). The normalization value is given in the title of each sub-figure. *all*, τ_all_ and *I*_all_, are errors where the squared errors of all features, all time constants and all current features are, respectively, added first before taking the root and mean. The depicted legend is valid in all sub-figures.

**Figure 3:**
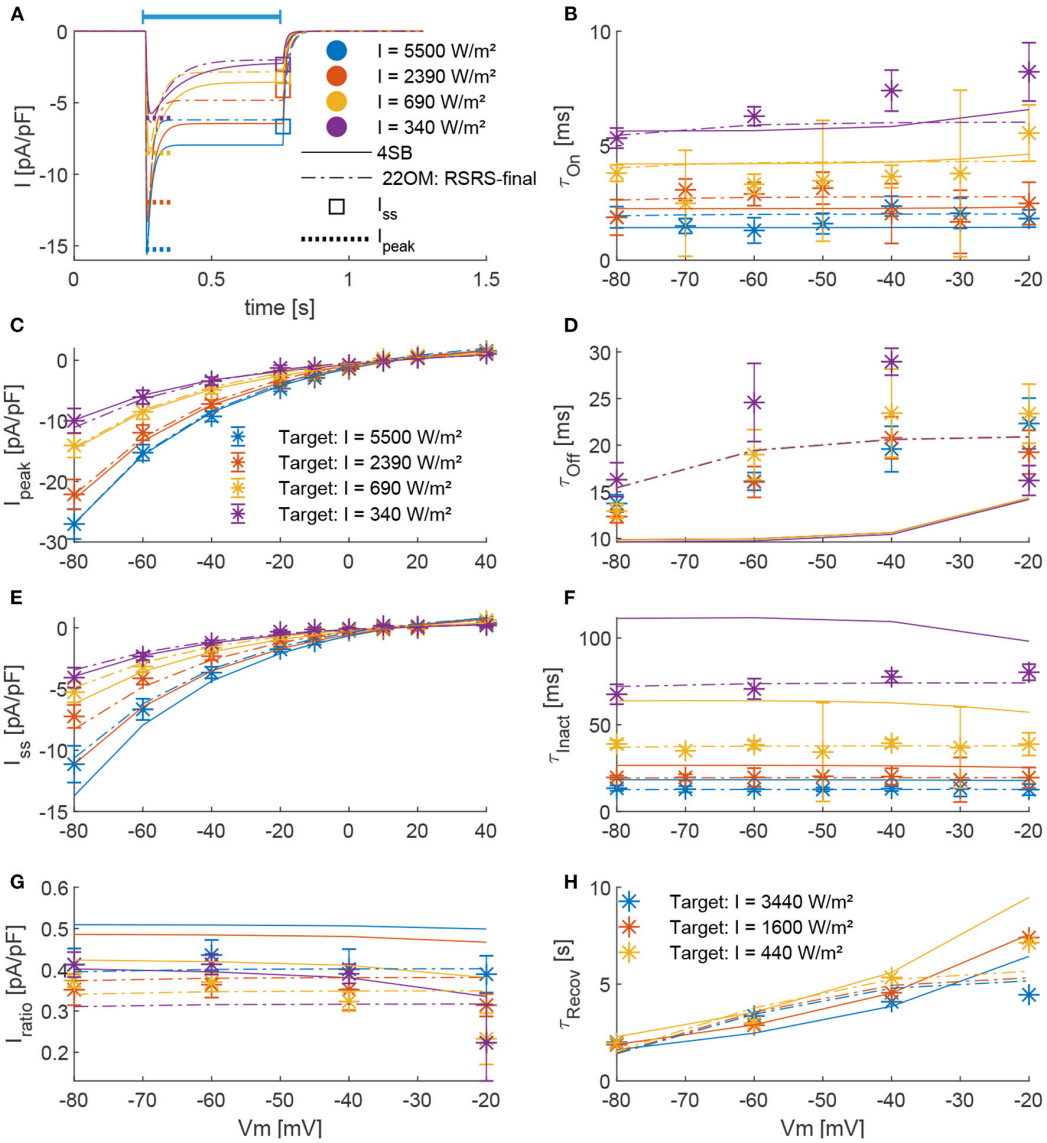
Comparison of model outcomes (4SB and 22OM: RSRS-final) with parameters obtained from experiments. **(A)**, The ChR2(H134R) current during a pulse of 0.5 s (indicated by blue bar) at a voltage clamp of –60 mV; according to the 4SB model (full lines) and 22OM model (dashed dotted lines). The colors indicate the applied intensity and are valid in **(A–G)**. The dotted line and square indicate respectively the experimental current peak and steady-state current at corresponding intensity and potential. **(B,D,F)**, Voltage dependence of respective τ_on_, τ_off_, and τ_inact_ across four irradiance levels. **(C,E,G)**, The current-voltage curves of the peak, steady-state and current ratio, respectively. The asterisks with errorbars indicate the experimental mean ± standard deviation. **(H)**, The recovery time constant as function of the membrane potential for three different irradiance levels as depicted in the plot.

The models' accuracy according to the four goodness-of-fit metrics Equation 22 are shown in Figure 2. Overall, a positive effect of the final optimization step can be observed. The largest impact is on the time constants, as expected. In the second step of the fitting procedure, the transition rate time-constants (τ_O_ and τ_R_) are approximated with a one on one relationship of the target features (see Equation 9 and 10). These approximations are true in case of high differences in order of magnitude. However, when the differences are smaller some cross correlations exist, for instance τ_R_ strongly affects τ_on_ as well, resulting in an underestimation of τ_O_. We denote that according to all metrics, the estimation accuracy of τ_on_ and τ_inact_ increases, however, at the cost of τ_off_. Also, a significant improvement is observed in case of τ_recov_. This deviation is due to the fact that the conditions Equation 11 are not fully met. Furthermore, an increased goodness-of-fit of the inactivation time constant can be observed in case of the RSRS vs PP fit. τ_R_ predominately defines both the inactivation and recovery time constant. In case of the PP fit, a separation of variables is applied where independence is assumed. However, as can be seen in Figures 3F,H, a more clear voltage dependency is present in τ_recov_ compared to τ_inact_. In other words, for low intensities (with high time constants as result) the potential effect is high while the effect is low for high intensities or small time constants. This interdependence is exactly obtained with the reciprocal addition scheme. The same, however less pronounced, can be observed in case of the activation and deactivation time constants (τ_on_ and τ_off_). Consequently, only the RSRS fit is used in further analysis.

Figure 3 shows a detailed comparison of the outcome of our model according to the RSRS fit and the 4SB model, vs. the experimentally determined target features. Overall, it can be observed that the proposed model performs at least as well as the 4SB model. Moreover, all features are well approximated. It can be seen that with the 4SB model, the steady-state value is overestimated in case of negative potentials (Figures 3A,E). However, a better representation is obtained for positive potentials, which explains the lower root-mean-squared normalized error (RMSNE, Figure 2B).

### 3.2. Neural Response in Regular Spiking Neuron

**Figure 4:**
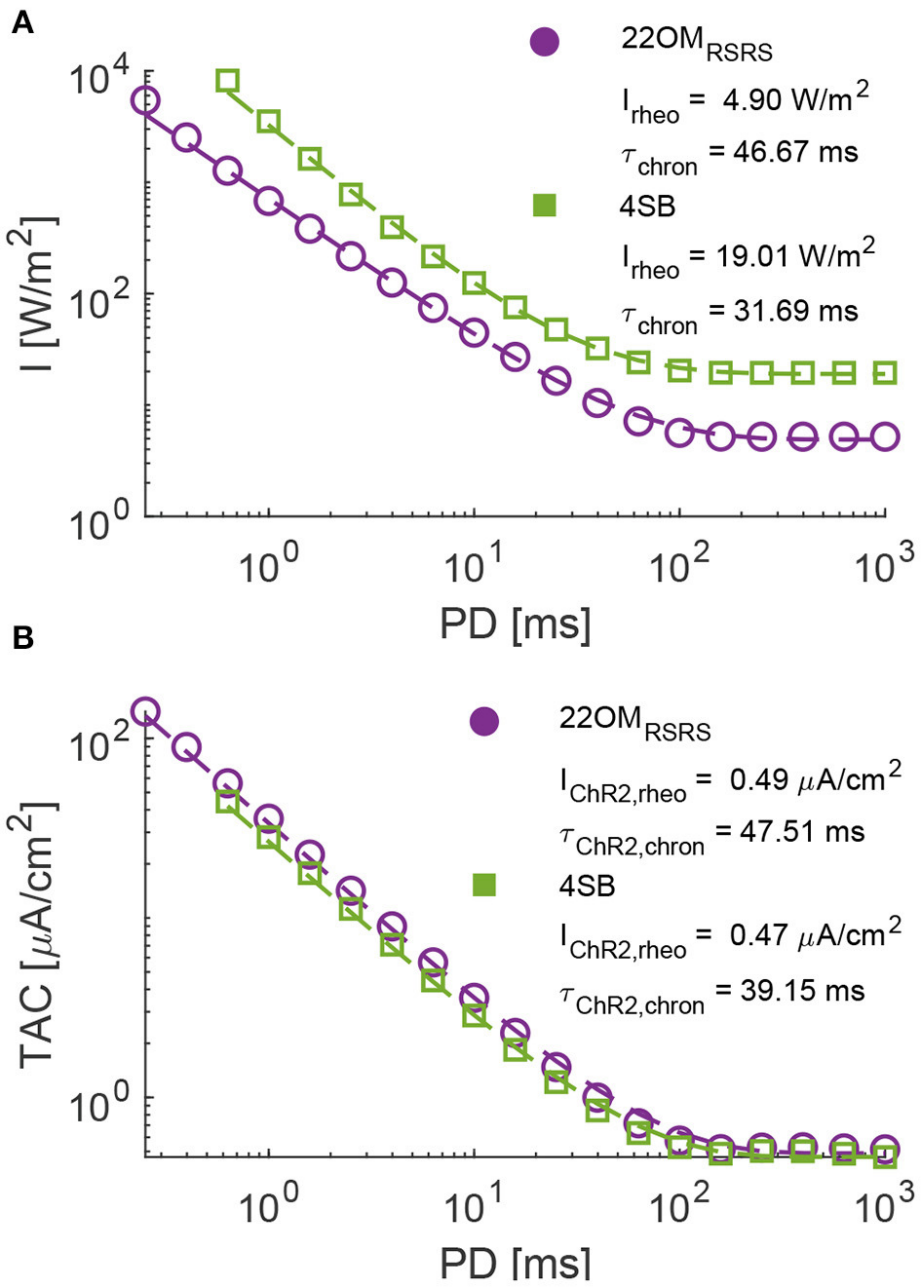
The strength duration curves (SDC) of the 22OM RSRS and 4SB model in a regular spiking neuron. **(A)**, Irradiance vs. pulse duration with a mapping (dashed line) of the SDC in **(B)** according to a power series. **(B)**, Temporal average current or average injected current vs. pulse duration. Dashed line represents the Hill-Lapicque model fit. The rheobase and chronaxie are depicted in the figures. The results of the 22OM and 4SB model are in purple and green, respectively.

To analyze the neural response, the strength duration curves (SDC) are determined of the proposed 22OM model with RSRS fit and the 4SB model in a regular spiking neuron, described in Pospischil et al. (2008). First, the Hill-Lapicque model fit is performed on temporal average current (TAC), as described in section 2.3. Very good fits were obtained for both models. The adjusted r^2^ (${\overset{-}{\text{R}}}^{2}$) of TAC vs. PD are 0.9961 and 0.9953 for the 22OM and 4SB model, respectively. The rheobase of the 22OM model (0.49 μA/cm^2^) is slightly higher than when the 4SB is used (0.47 μA/cm^2^). Also the chronaxie is higher (47.51 ms vs. 39.45 ms). Consequently, according to the 4SB model for any pulse duration, less charge is injected optogenetically to excite a regular spiking neuron via a ChR2(H134R) opsin. The difference between the models can be attributed to the difference in deactivation time constant (τ_off_). This is higher in the 22OM model resulting in a slower closing mechanism and thus increased current injection after the AP. A good cell-type-specific empirical mapping of TAC to irradiance was obtained as well (Equation 25), with ${\overset{-}{\text{R}}}^{2}$ values of 0.9449 (22OM) and 0.9638 (4SB). The parameter values are respectively, a = 8.18, b = 1.26 and c = 1.68, and a = 22.30, b = 1.51 and c = 12.32 in case of the 22OM and 4SB mapping. The lower ${\overset{-}{\text{R}}}^{2}$ of the 22OM mapping resulted also in a slightly lower value of 0.9298 for the irradiance to PD curve while this is 0.9509 in case of the 4SB fit. Based on the mapping parameters and Figure 4, it can be seen that lower intensity level results in higher injected currents when the 22OM model is used. Indeed, extrapolation of the model fit to low intensities results in higher open probabilities than for the 4SB model, hence the difference in irradiance rheobase of 4.90 W/m^2^ vs. 19.01 W/m^2^. Based on the higher peak values for high intensities in case of the 4SB model, one could expect convergence of the irradiance SDCs. However, due to the slow activation kinetics, the peak value is not reached at small pulse durations. Even though the activation time constant is overall higher for the 22OM model (Figure 3B), the bi-exponential current rise due to the extra state variable [τ_ChR2_·*dp*/*dt* = *S*0(*I*) − *p*, a time-dependent function reflecting the probabilistic, non-instantaneous response of the ChR2-retinal complex to light Williams et al., 2013] in the 4SB model results in a lower current value at the end of the pulse.

### 3.3. Computational Speed

**Figure 5:**
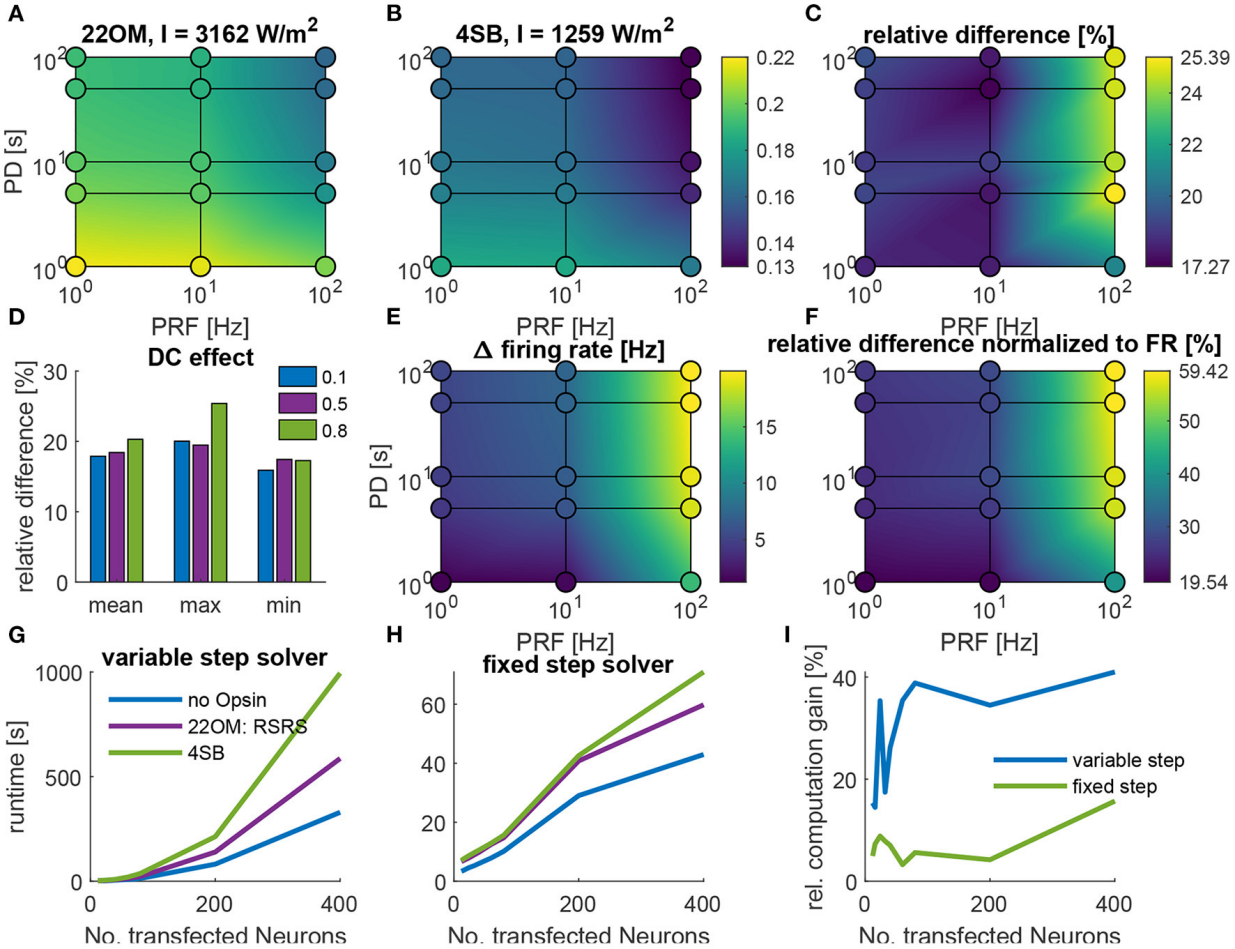
The computational speed of optogenetic neuromodulation in a regular spiking (RS) neuron and sparse Pyramidal-Interneuron-Network-Gamma (sPING). **(A–F)**, Simulation speed, i.e., simulation time/runtime, for different stimulation protocols with varying pulse duration (PD) and pulse repetition frequency (PRF) in a regular spiking neuron, described by Pospischil et al. (2008). **(A)**, The absolute simulation speed with the 22OM-RSRS fit. **(B)**, The simulation speed with the 4SB model. Colorbar is valid for **(A)** and **(B)**. **(C)**, The relative difference, i.e., (22OM-4SB)/4SB. **(D)**, The effect of the duty cycle on the simulation speed. **(E)**, The difference in firing rate in case of the 22OM model vs. 4SB. **(F)**, The relative difference of simulation speed normalized to the firing rate. **(G–I)**, Runtime of a continuous 300 ms optical pulse in the sparse Pyramidal-Interneuron-Network-Gamma (sPING), with increasing number of transfected neurons. **(G)**, Runtime with a variable step solver. **(H)**, Runtime with a fixed step solver. **(I)**, Relative computation gain, i.e., -(22OM-4SB)/4SB. The used intensities are shown in the titles of **(A,B)**, which give rise to a 100 Hz firing rate (see section 2.3).

The proposed model in this study contains only two differential equations, which is 50% less in comparison with the 4SB model. Consequently, a reduction of the computational time is expected. Figures 5A–F summarizes the computational speed for different stimulation protocols in a regular spiking neuron. This for fixed irradiances (22OM: 3162 W/m^2^ and 4SB: 1259 W/m^2^) set to a value that elicit a firing rate of 100 Hz, as described in section 2.3. Figures 5A–D show an overall increase of the computational speed in favor of the 22OM model, with a maximum of 25% for high frequency and duty cycle stimulation. On average the relative difference of the simulation speeds, i.e., simulation speed with 22OM minus simulation speed with 4SB with respect to the latter, is about 20%. Because the simulations were solved using a variable step solver, the difference in firing rate could distort the effective simulation speed, as during an action potential a smaller timestep is selected. Therefore, the relative difference of the simulation speed normalized to the firing rate is depicted as well, with an increase of the gain to 60% as result. The runtime vs. number of transfected neurons is depicted in Figures 5G–I. The simulation outcomes were the same with the variable and fixed step solver, validating the solver settings. Moreover, the firing rate was equal for both opsin models, hence no normalization was necessary. A clear reduction can be observed when the 22OM model is selected instead of the 4SB model, both with a fixed and variable step solver. The time gain by using the proposed model is 15% (5%) in case of 12 neurons and goes up to 40% (15%) and rising when 400 transfected neurons are included with a variable (fixed) step solver.

### 3.4. Versatility of the Proposed Model

Finally, we address the versatility of the proposed model and the fitting procedure. Due to the increasing number of possible opsins, it is favorable that their kinetics can be correctly modeled and a fit is easily obtained without preliminary knowledge. To this end, we applied our fitting procedure to experimental data of a MerMAID opsin, which has unlike classical ChR2 a very strong desensitization (Oppermann et al., 2019). Starting from the photocurrent traces, the target features had to be extracted first. Next the parameter space was defined. The rectification function was omitted because this was not observed in the experimental data. Aside from this, the lower bound and initial condition of only the third parameter of *R*_∞_ was altered (Table 1). This straight forward adjustment was made due to the strong desensitization. The weights of the cost function were set to *w*_peak_ = 0.04, *w*_ss_ = 1, *w*_ratio_ = 250, *w*_on_ = 10000, *w*_inact_ = 10000, *w*_off_ = 10000, *w*_recov_ = 10, again to level the errors to the same order of magnitude. Because no saturation of the current was observed at high intensity levels a constraint: *O*_∞_(*I, V*) < 0.5 for *I* ≤ 4000W/m^2^, was added.

**Figure 6:**
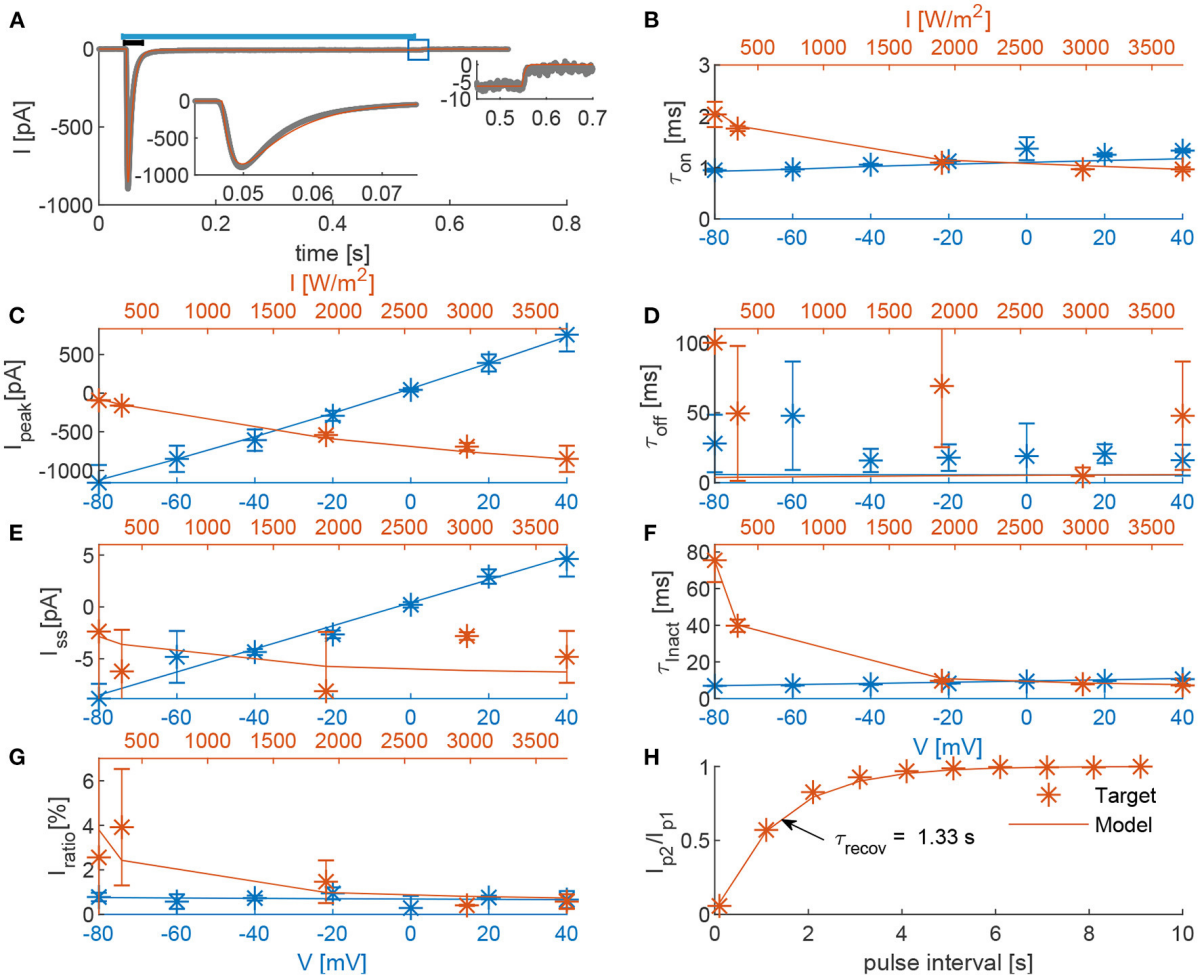
Comparison of the 22OM-Mermaid (final fit) model outcomes and experimental data. **(A)**, In gray, the photocurrent of a voltage clamp experiment during a 0.5 s continuous illumination with an intensity of 3,734 W/m^2^ (indicated with blue bar on top) (Oppermann et al., 2019); In red, the corresponding model outcome. Left inset is a zoom of the current peak (0.045–0.075 s, indicated with black bar). Right inset is a zoom of the current deactivation (0.45–0.7 s, indicated with a blue square). **(B–G)**, The voltage dependence of the target features (τ_on_, *I*_peak_, τ_off_, *I*_ss_, τ_inact_, and *I*_ratio_) at an irradiance of 3,734.4 W/m^2^ is shown in blue. The light dependence at a holding potential of –60 mV is depicted in red. **(H)**, Ratio of the peak currents in response to a two-pulse stimulation protocol at –60 mV and 3,734 W/m^2^ as function of the inter-pulse interval. The recovery time (the interval time necessary to have a ratio of 63%), is indicated with a black arrow.

The result of the fit is shown in Figure 6. The parameters of the final and intermediate fit are summarized in Table 1. The model here is with a double product combination of the time constant dependencies. Because the recovery time constant was only determined under one condition, there is no evidence on the interdependence of the variables. This is also supported by the small voltage dependence of the (de)activation time constants. Overall, it can be stated that a good fit is obtained as all kinetics are expressed correctly. Only, the deactivation time constant seems to be underestimated. This is a consequence of the constraint in Equation 21, which ensures a current decay back to baseline after optical stimulation. Due to its strong desensitization, *R*_∞_ has to be small, thus inducing an upper limit on τ_O_(0, *V*), which defines the deactivation time constant. The trade off is justified due to the higher uncertainty of the deactivation time constant (see Figure 6D). Moreover, the overall effect is expected to be low as can be seen in the right inset of Figure 6A.

## 4. Discussion

The proposed double two-state model structure for the modeling of opsins appears to be a good alternative to the computationally more expensive four state Markov, non-instantaneous models. All features are represented, with even some improved fit accuracy in comparison with a four state Markov variant. Furthermore, with the proposed fitting procedure, we were able to fit two opsins, ChR2(H134R) and MerMAID. Although the prominent difference of the mutants kinetics, the fitting procedure allowed us to get these fits with only minor adjustments of the parameter space and constraints. Therefore, creating the possibility for autonomous model fitting based on photocurrent traces. Moreover, a good fit is obtained within an acceptable time frame, due to the absence of differential equations in the fitting procedure, which is not achievable in case of a four state Markov model. The intermediate fit is obtained within 3 h, while the final fit always flagged the time limit of 24 h. Increasing the limit improves the fit accuracy but only small changes were observed. Fine tuning of the optimization settings, such as number of particles or tolerances, could reduce the training error even more. However, this is out of the scope of this study.

The proposed model is an empirical model. The fit is performed on a limited dataset thus extrapolation should be treated with care. This is clear from the neural response results in section 3.2. Although both the 4SB and our model were fit to the same experimental data, a clear discrepancy between the fitted rheobase is observed [4.90 W/m^2^ (22OM) vs. 19.01 W/m^2^ (4SB)]. Unlike the chronaxie where the difference can be attributed to the model's structure, the difference in rheobase is due to the discrepancy between opening rates after extrapolation to low intensities, attributed to the fit and intensity dependence chosen in each model. More experiments are required in order to validate this.

The dependencies chosen here are all, except the rectification, sigmoidal. Therefore, they are all bounded and monotonic. This is in accordance with a channel's behavior, i.e., increased and faster opening at higher intensities but limited to an open probability of one. We opted for a biphasic logistics function for τ_R_(*I*) modeling. This is in agreement with the hypothesis of the necessity of two light dependent rates [(R → S) and (S → R), see section 2.1] and the second and third photochemical pathways described by Kuhne et al. (2019) (Figure 1). Other functions were tested, e.g., weibull or asymmetric logistics with double intensity dependence, however no improvement was observed. Initially, separation of variables was assumed to suffice due to the lack of experimental evidence of complex channel interdependence of both irradiance and potential of each feature separately. However, due to the models structure, τ_on_ and τ_off_ share the same voltage dependency, as well as τ_inact_ an τ_recov_. The voltage dependence of τ_recov_ and τ_off_ was clearly more pronounced in the experimental data of the ChR2(H134R) mutant. Therefore, the reciprocal addition Equation 16 was tested as alternative, resulting in an improved fit accuracy. However, this only scales down the voltage dependent effect on τ_on_ and τ_inact_ while the same relationship is maintained. The necessity of more complex relationships could be investigated in future work as well as the need for voltage dependence of the rate functions steady-state values (*O*_∞_ and *R*_∞_), which was omitted in this study.

Currently the model incorporates voltage and irradiance dependence. Studies have however shown the importance of pH on the channel kinetics in many opsins. Furthermore, ion concentrations have an impact on the reversal potentials and current rectification (Berndt et al., 2010; Stehfest and Hegemann, 2010). Schneider et al. (2013), postulated a model based on the kinetics of multiple ion species interacting with the channel, with an improved representation of the current rectification (Schneider et al., 2013; Williams et al., 2013). While the photocurrent properties are unaffected by pH-changes, the MerMAID photocurrent is strongly dependent on the Cl^−^ concentrations. The fit performed here was on experimental data recorded with an extracellular Cl^−^ concentration of 150 mM and intracellular Cl^−^ of 120 mM, explaining the depolarizing currents (negative sign in Figure 6) as an anion conducting channel. By changing the extracellular concentration to 10 mM, the channel's reversal potential is shifted to the reversal potential of Cl^−^. (The concentrations are exchanged with respect to a conventional neuron, where the typical intracellular and extracellular concentrations are 10 and 120 mM, respectively. This explains the experimentally measured depolarizing currents (negative sign), while one would expect hyperpolarizing currents (positive sign) from a Cl^−^ conducting channel.) Evidence of the Cl^−^ effect on channel kinetics is still absent but further experiments are needed (Oppermann et al., 2019). Consequently, the model fit shown here can be used in computational studies but the reversal potential should be adjusted accordingly.

With the current model structure, the model responds instantaneously to light (see left inset Figure 6A). With the 4SB model this is circumvented by adding a extra state variable with a time constant of 1.5 ms. It is clear that for long (PD >> τ_on_) continuous pulses its effect is negligible, as activation is dominated by the activation time constant. However, with short bursts or pulses, this non-instantaneous activation becomes prominent as observed in section 3.2. In future work, it could therefore be interesting to incorporate this non-instantaneous response. This could probably be obtained by adding an extra state variable, as performed with the 4SB model, however at the cost of the computational speed. Another possibility is to raise the open state, O(t), to a higher power, smoothing the transition but without irradiance control. Modification of the model's structure could be circumvented by gradually increasing the intensity, instead of applying a rectangular pulse.

## 5. Conclusion

To facilitate computational studies in the field of optogenetics, we proposed a double two-state opsin model structure as alternative to the conventional three and four state Markov models. In the proposed model, the second state pair represents the conductance regulation due the dark-light adaptation. With this model type, a reduction in complexity is obtained resulting in only two differential equations compared to four in case of the preferred, non-instantaneous four state Markov models used for opsin modeling. With the provided fitting procedure, autonomous model fits of two distinctive opsins (ChR2(H134R) and MerMAID) were obtained. Both model fits were performed within an acceptable time frame thanks to the absence of differential equations and parameter space reduction associated with the multi step approach. Moreover, both models are able to represent the experimental data with great accuracy. Due to the model's structure, there is, however, an instantaneous response to light, overestimating the injected current at very short pulses (< τ_*on*_). Furthermore, pH and ion concentration dependence are not incorporated. In its current state with only two differential equations, the computational speed is increased up to 25% in a regular spiking neuron and up to 40% in a network of 400 transfected neurons.

## Data Availability Statement

The original contributions presented in the study are publicly available. Requests to access these datasets should be directed to Ruben.Schoeters@UGent.be.

## Author Contributions

RS, TT, LM, WJ, RR, and ET contributed to the study design. RS performed all simulations. RS, TT, and ET prepared the manuscript. All authors read and approved the final manuscript.

## Conflict of Interest

The authors declare that the research was conducted in the absence of any commercial or financial relationships that could be construed as a potential conflict of interest.
